# Can we well assess the relative efficacy and tolerability of a new drug versus others at the time of marketing authorization using mixed treatment comparisons? A detailed illustration with escitalopram

**DOI:** 10.3402/jmahp.v3.26776

**Published:** 2015-09-24

**Authors:** Pierre-Michel Llorca, Christophe Lançon, Mélanie Brignone, Caroline Painchault, Benoit Rive, Mondher Toumi, Clément François

**Affiliations:** 1 Centre Hospitalier Universitaire, Université d'Auvergne, Clermont-Ferrand, France; 2 Hôpital Sainte-Marguerite CHU, Université d'Aix-Marseille, Marseille, France; 3 Lundbeck SAS, Paris, France; 4 Keyrus Biopharma, Paris, France; 5 Creativ Ceutical, Paris, France; 6 Lundbeck A/S, Deerfield, IL, USA

**Keywords:** major depressive disorder, mixed treatment comparisons, antidepressants

## Abstract

**Objective:**

To assess the variation of relative efficacy and tolerability of an antidepressant versus others based on both pre-marketing (registration studies) and post-marketing studies versus pre-marketing studies only in patients with major depressive disorder.

**Methods:**

The relative efficacy and tolerability of antidepressants was assessed by mixed treatment comparisons (MTCs) using data acquired over two time periods: before registration of the reference drug escitalopram (1989–2002) and up to 5 years later (1989–2007). Ranking probability outputs were presented for efficacy, using change from baseline to 8 weeks on Montgomery–Åsberg Depression Rating Scale total score, and tolerability, using withdrawals due to adverse events.

**Results:**

The relative efficacy and tolerability of some selected antidepressants, including escitalopram, varied considerably over the two time periods. The improved relative efficacy and tolerability of escitalopram over time, compared with citalopram, was demonstrated by greater separation of ranking probability curves for efficacy and tolerability. In 2002, escitalopram ranked low with 13.9% and 5.1% probability of being in the top four antidepressants’ relative efficacy and tolerability, respectively. In 2007, ranking probabilities for relative efficacy and tolerability of escitalopram increased to 52.5% and 82.1%, respectively.

**Conclusions:**

Time of marketing authorization may not be the most appropriate time to evaluate the relative efficacy and tolerability of a new antidepressant based on MTC approach due to the asymmetry of information between new and older compounds. However, the first evaluation of relative effect of a new drug for health technology assessment recommendations is commonly done at this time. Re-evaluation of a drug several years after its launch is likely to provide a more accurate indication of its relative efficacy and tolerability.

One prerequisite before placing a new product on the market is the need for clear evidence that it meets certain essential requirements, including a favorable benefit/risk ratio. For example, for the treatment of major depressive disorder (MDD), any new medicinal products are developed according to defined criteria found in regulatory guidance (1). To permit an adequate evaluation of a new drug, registration studies are typically placebo-controlled and may also include an active reference drug to confirm assay sensitivity. Registration studies are also required to use sufficient duration of treatment, stringent inclusion and exclusion criteria, and validated rating instruments to assess drug efficacy (e.g., for depression the two recognized scales are the Hamilton Depression Rating Scale [HAM-D] and the Montgomery–Åsberg Depression Rating Scale [MADRS]) (1–3).

Clinical data obtained post-marketing authorization (MA) are intended to supplement or expand registration trial data. Such post-marketing studies are critical to update clinical benefit risk evaluation throughout the life cycle of the drug and to record the long-term safety of the drug. Therefore, post-registration studies are expected to differ from pre-approval studies in design, population enrolled, and so on.

To determine the relative efficacy of a therapeutic class, network meta-analyses (NMAs) can be conducted to generate comparative evidence from multiple randomized clinical trials (RCTs) assessing the same efficacy outcomes and indication, but involving different interventions (4, 5). However, these evaluations usually include both direct and indirect comparisons based on different types of studies (pre- and post-MA studies). The results of these analyses often have to be interpreted with caution as the direct and indirect evidence are not always consistent one with each other.

The objective of this study is to assess the variation of relative efficacy and tolerability of an antidepressant, escitalopram, versus others based on both pre-marketing (registration studies) and post-marketing studies versus pre-marketing studies only in patients with MDD. The present study reports the results of a comparison between the selected antidepressants over two time periods using a mixed treatment comparison (MTC) method (5, 6).

## Methods

The first analyses incorporated only registration studies up to the MA date of escitalopram (an allosteric selective serotonin reuptake inhibitor [SSRI]) which was chosen as the reference antidepressant.

The second analyses included both registration and post-marketing studies in the same indication that were published up to 5 years later.

Therefore, the current settings were similar to the conditions of a health technology assessment (HTA) evaluation.

### Time periods

Two time periods, from 1989 to 2002 and from 1989 to 2007, were used for the analysis of the relative efficacy and tolerability of antidepressants. The first time period included studies up to 2002, the year of the MA for escitalopram in both the United States and Europe. In practice this means that only registration studies were considered for escitalopram on this first time period, while both registration and post-marketing studies were included for the other antidepressants as they received a marketing authorization earlier. The second time period included additional studies that were published up to 5 years post-approval and ended in 2007, as reassessment of drugs by HTA authorities are often conducted after this time period.

### Study selection and antidepressants evaluation

In order to define a homogeneous pool of treatments between the two time periods only the following new generation antidepressants for which the MA was obtained before 2002 were included: citalopram, escitalopram, fluoxetine, fluvoxamine, milnacipran, mirtazapine, paroxetine, reboxetine, sertraline, and venlafaxine (pooled IR and XR) Placebo was also considered. Clinical studies were retrieved from a systematic review performed on MDD. Studies were identified by searching MEDLINE, Embase, Cochrane Central Register of Controlled Trials, and PsychINFO. The following conference proceedings were hand searched: American Psychiatry Association (APA), European College of Neuropsychopharmacology (ECNP), International College of Neuropsychopharmacology (CINP), New Clinical Drug Evaluation Unit (NCDEU) with the following public and company registries: Clinicaltrials.gov, Clinicalstudyresults.org, Lundbecktrials.com, Forestclinicaltrials.com, Lillytrials.com, Novartis register, EMEA websites for retrieving the European Public Assessment Reports (EPAR), and FDA websites.

Non-English publications were excluded from the systematic review. Studies were also excluded if they did not report subgroup data for depression in an adult population (in case of larger patient selection) or did not report any of the outcomes of interest, or if they examined the continued use of antidepressants for relapse or recurrence prevention, or if they included <30 patients per treatment group.

Only treatment arms including approved doses of the selected antidepressants and placebo were included in the analyses (Table 1). If several approved doses of an antidepressant were included in a given RCT, doses were pooled together and treated as a single treatment arm (4).

**Table 1:** Recommended doses and approval dates of antidepressants included in the present analysis

	Approved dose (mg/day)^a^	Date of marketing authorization		Number of RCTs in the MTC analysis of MADRS change from baseline		Number of RCTs in the MTC analysis of withdrawals rate due to AEs	
			
		EU	US	1989–2002	1989–2007	1989–2002	1989–2007
Citalopram	20–60	30/01/1989 (Denmark)	17/07/1998	7	14	8	14
Escitalopram	10–20	14/12/2001 (Sweden)	14/08/2002	3	18	3	18
Fluoxetine	20–80	1989 (UK)	29/12/1987	22	28	37	49
Fluvoxamine	50–300	1983 (Germany)	05/12/1994	3	3	10	12
Milnacipran	50–200	06/12/1996 (France)	Not approved in MDD	3	4	3	4
Mirtazapine	15–45	16/03/1994 (The Netherlands)	14/07/1996	5	6	7	11
Paroxetine	20–50	11/12/1990 (UK)	29/12/1992	15	21	22	41
Reboxetine	8–12	10/04/1997 (UK)	Not approved in MDD	2	2	2	2
Sertraline	50–200	19/11/1990 (UK)	30/12/1991	6	10	15	24
Venlafaxine	75–375	01/06/1994 (France)	28/12/1993	17	22	16	24

AEs: adverse events, EU: Europe, MADRS: Montgomery–Åsberg Depression Rating Scale, MDD: major depressive disorder, MTC: mixed treatment comparison, RCT: randomized controlled trial, US: United States.

a Data sources: Summary of Product Characteristics and US label.

### Outcomes

Efficacy was evaluated as the change from baseline to 8 weeks (time window for assessment visit from 6 to 12 weeks) in the MADRS total score, which was the primary outcome in most of the escitalopram and citalopram studies considered above. Any studies without MADRS outputs were not used, including those that only reported outcomes based on the HAM-D. Tolerability was defined as the proportion of patients who withdrew from the study due to adverse events (AEs) (study duration between 6 and 12 weeks), out of the total number of patients randomly assigned to each antidepressant or placebo and who received at least one dose of IMP.

### Imputation methods

If any values were not reported in the original data source, queries were made to find the data. In addition, imputation rules were defined for cases where data were missing for the year of publication or the standard deviation (SD) of the change from baseline to 8 weeks on the MADRS. Missing years of publication data were imputed by the date of the clinical report plus 1 year or the date of study completion plus 2 years. Missing SD values were either recalculated from the standard error (SE) or 95% Credible Interval (CrI) values, or imputed by 10 (value defined by the observed mean of the SD across studies and treatment arms). When necessary, the change from baseline was recalculated as the difference between endpoint and baseline values (SD imputed by 10, corresponding to the rounded value of the average of the SDs observed across all studies and treatment arms). This imputation was performed before the pooling of doses so that differences between doses results in additional variability.

### Data analysis

MTCs (5, 6) were performed to assess the relative efficacy and tolerability of the selected antidepressants based on available RCT data from the two time periods. A Bayesian NMA with random effect was performed using a linear model with normal likelihood distribution for the MADRS change from baseline and a logistic model with binomial distribution for withdrawals due to AEs. The random effect model specification was chosen as it provides conservative estimates of variance and as such reduces the risk of spurious findings compared to the fixed effect specification. Vague priors were assigned to the parameters of interest: normal distributions with mean 0 and variance 10,000 for the baseline and treatment effects of the trials, and uniform distribution between 0 and 5 for the between-trial SD. Initial values were randomly drawn independently for three parallel chains. As this process is iterative, a thin rate of two was applied in order to reduce auto-correlation between consecutive draws. The Bayesian analysis consisted of 40,000 draws with a burn-in of 10,000 in order to ensure convergence (so that the draws used in the analyses actually inform on the uncertainty of parameters of interest and not on convergence). Outcomes were entered for every treatment group of each study and relations between the relative efficacy indicators (difference between treatments or odds ratios [OR]) across studies were specified in order to estimate all possible pairwise comparisons between treatments. This method combined direct and indirect evidence for any given pair of treatments. Based on the rank of treatments in each Bayesian draw, the probability for each treatment to have a given rank and the mean ranks were estimated.

An analysis of the relative efficacy and tolerability of the antidepressants was performed for the two embedded time periods (1989–2002 and 1989–2007). Outputs generated from these analyses were presented as ranking probabilities for MADRS change from baseline and for withdrawal rate due to AEs, respectively. Ranking probability curves for escitalopram (the active *S*-enantiomer of citalopram) and citalopram (a racemic mixture of *S*- and *R*-citalopram, originally launched in 1989) were examined more thoroughly (7).

A comparison of the relative efficacy and tolerability was also made for all antidepressants meeting the inclusion criteria for analysis and placebo. Difference to placebo, mean ranks, and probability of being ranked among the top four antidepressants are also presented.

Software: analyses for the MTCs were run with WinBUGS^®^ version 1.4, using standard programs developed by Bristol university. Programs can be provided upon request.

All analyses were performed by one statistician and quality control was conducted by another.

Results from analyses were then compared and any discrepancies in the analyses were resolved by program examination by the statisticians.

## Results

**Fig. 1:**
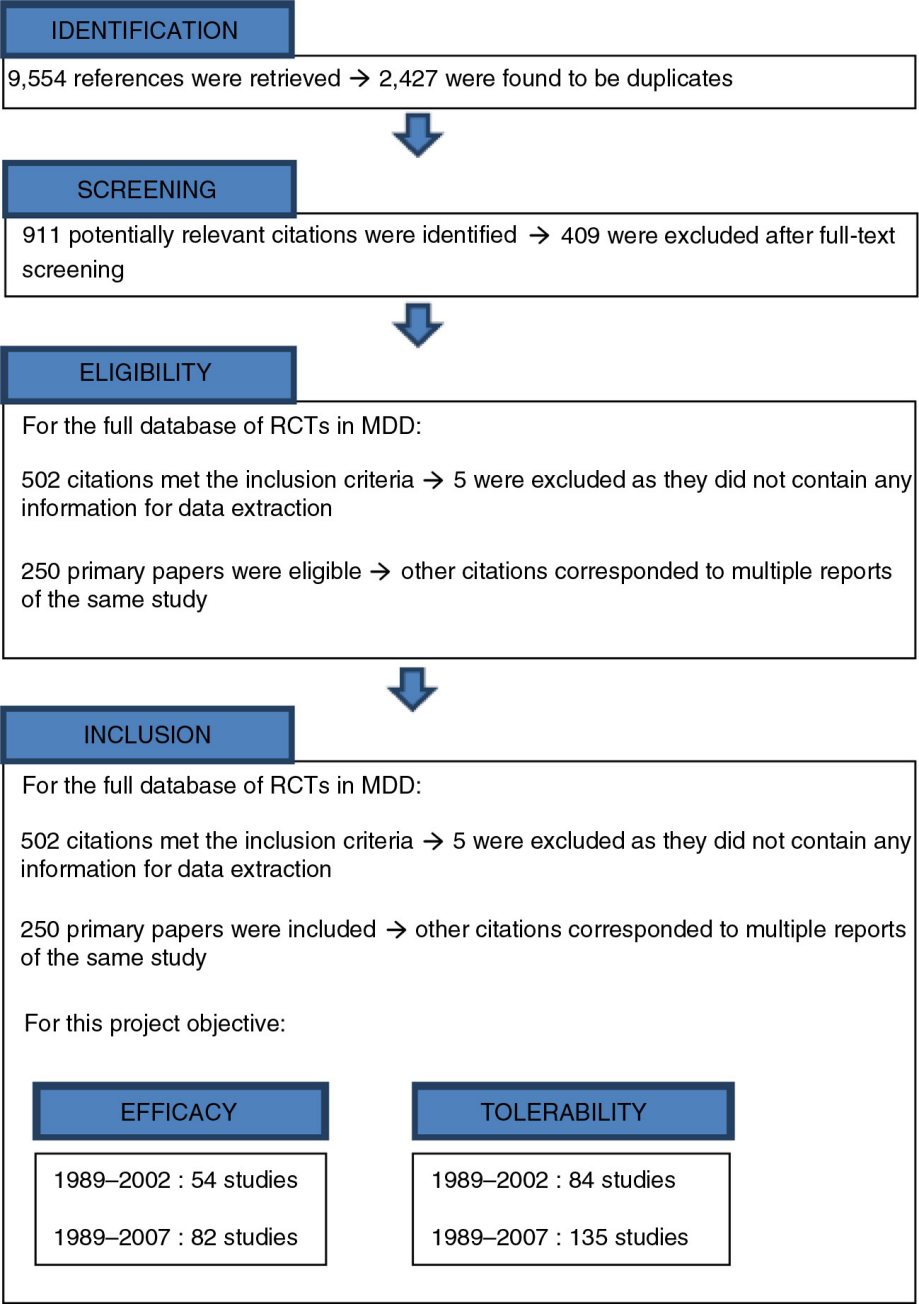
Study flow chart.

The number of RCTs selected from the database and included in the MTC analysis of efficacy and tolerability for each antidepressant and each time period (1989–2002 and 1989–2007, respectively) is detailed in Table 1 and a flowchart is presented in Fig. 1.

### Rankings for efficacy outcomes over the two time periods

**Fig. 2:**
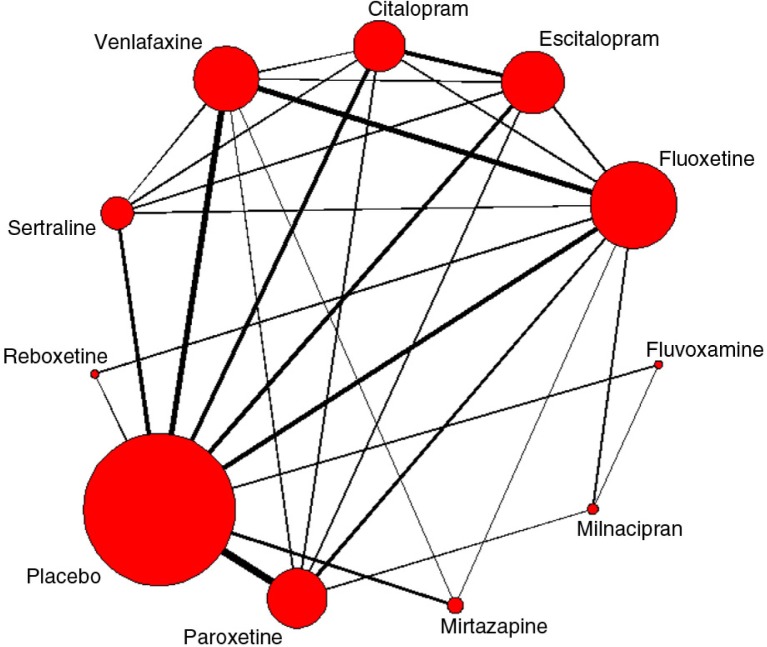
Network diagram of studies included in the efficacy analyses (time period of 1989–2007: 82 studies - see Appendix A for the complete list of included studies). The thicker the lines between two treatments, the more studies linking the two treatments.

Fifty-four studies were selected for the efficacy evaluation (as assessed by the change from baseline in MADRS reported at 8 weeks) for the time period 1989–2002 and 82 studies for 1989–2007 (Fig. 2). Within the first time period, mirtazapine, venlafaxine, and paroxetine ranked highest overall (mean ranks of 2.00, 2.64, and 3.38, respectively), while fluvoxamine ranked lowest among active treatments (mean rank 9.98), just above placebo (mean rank 10.69). A comparison of findings for the two time periods revealed some ranking probability changes between 2002 and 2007. However, mirtazapine and venlafaxine still ranked highest in terms of efficacy (mean ranks of 1.86 and 2.71, respectively) in 2007. Fluvoxamine continued to rank lowest for efficacy (mean rank 9.97), with a mean rank value for placebo of 10.64.

**Fig. 3:**
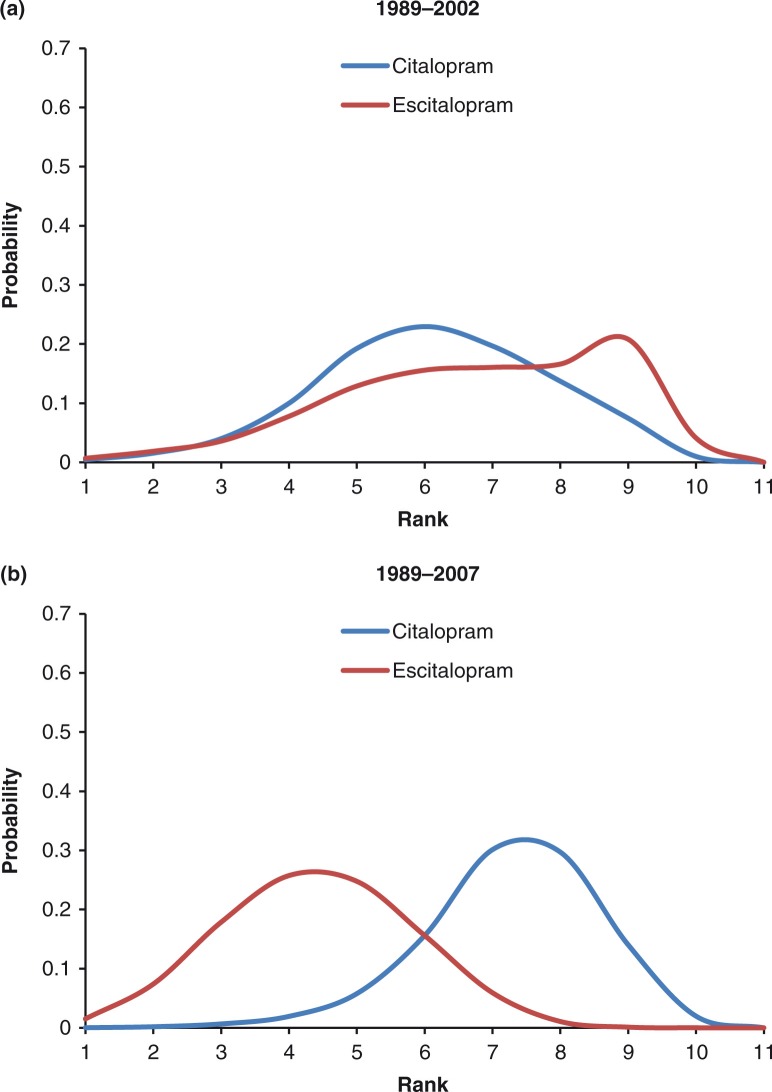
Ranking probabilities for escitalopram and citalopram for MADRS change from baseline to 8 weeks.

Focusing more precisely on escitalopram and citalopram, their MADRS change from baseline ranking probability curves were mostly overlapping in 2002 (Fig. 3a), while a much clearer separation in favor of escitalopram was observed in 2007 (Fig. 3b). MADRS change from baseline of escitalopram compared to placebo increased from −3.39 (95% CrI: −5.10 to −1.69) in 2002 to −3.77 (95% CrI: −4.66 to −2.90) in 2007. Treatment mean ranks for MADRS change from baseline at 8 weeks were 6.78 and 6.14 in 2002, compared to 4.42 and 7.27 in 2007 for escitalopram and citalopram, respectively.

### Rankings for tolerability outcomes over the two time periods

**Fig. 4:**
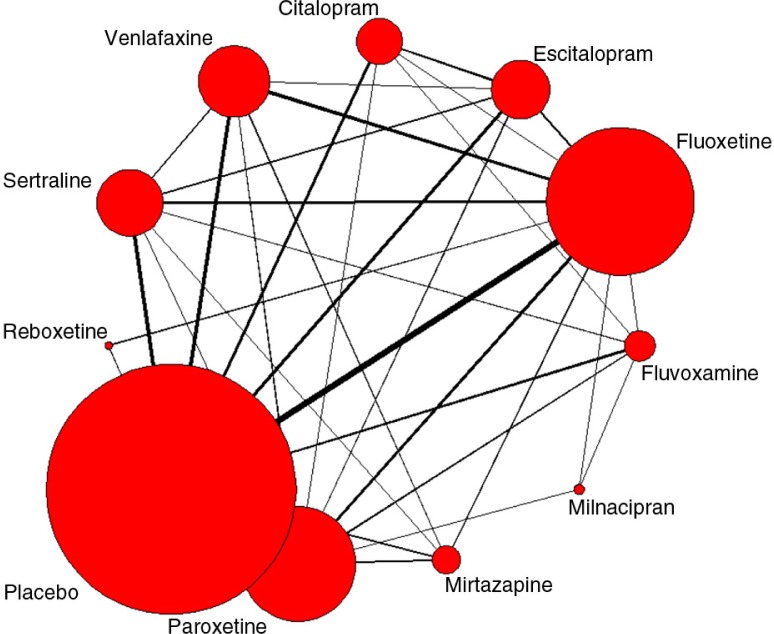
Network diagram of studies included in the tolerability analysis (time period of 1989–2007: 135 studies - see Appendix B for the complete list of included studies). The thicker the lines between two treatments, the more studies linking the two treatments.

Eighty-four studies were selected for the tolerability evaluation (as assessed by the withdrawals rates due to AEs reported at 8 weeks) for the time period 1989–2002 and 135 for 1989–2007 for the antidepressants of interest (Fig. 4). In 2002, milnacipran ranked highest for tolerability with a mean rank of 2.12 compared with 1.39 for placebo, while escitalopram ranked lowest at 9.21. However, differences between drug tolerability rankings were seen for 1989–2007 data, with both milnacipran and escitalopram ranked high. Mean ranks were 3.14 for milnacipran, 3.42 for escitalopram, and 1.11 for placebo.

**Fig. 5:**
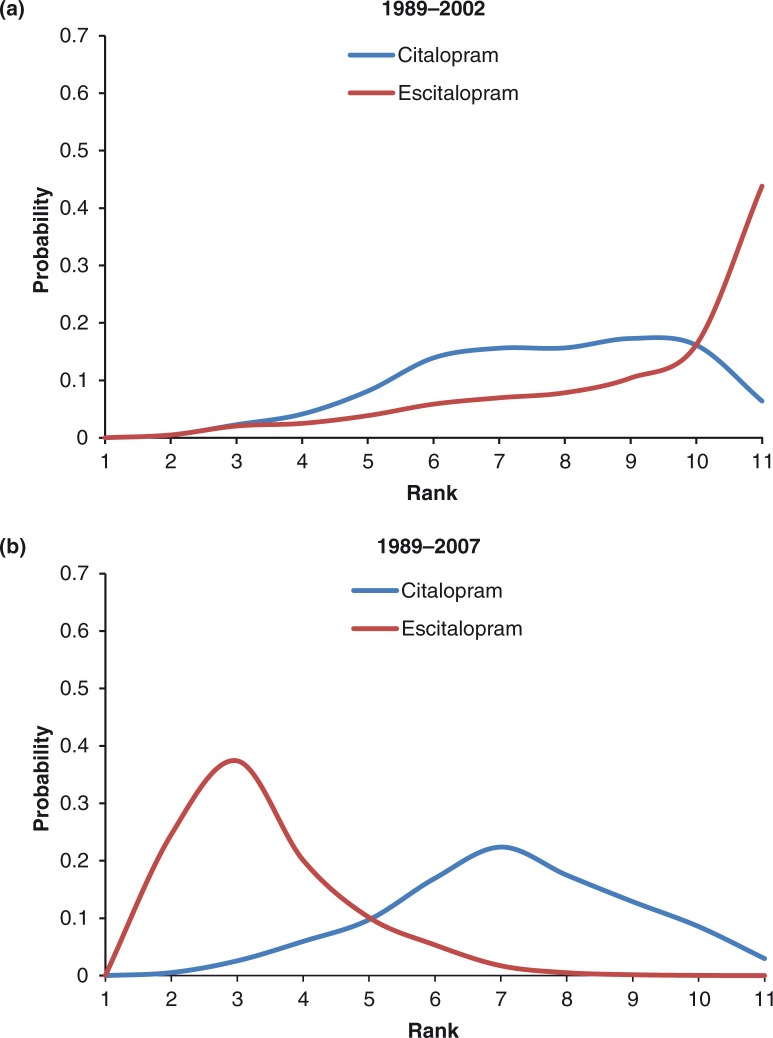
Ranking probabilities for escitalopram and citalopram for withdrawal rate due to adverse events at 8 weeks.

When escitalopram and citalopram were compared over the two time periods, ranking probability curves showed a clear differentiation between escitalopram and citalopram tolerability in 2007, in favor of escitalopram (Fig. 5b). In contrast, the ranking probability curve for escitalopram based on data up to 2002 provided clear evidence of non-separation of escitalopram's improved tolerability relative to citalopram (Fig. 5a). Differences for escitalopram compared to placebo in the log-OR for dropout rates decreased from 1.29 (95% CrI: 0.59 to 2.02) in 2002 to 0.56 (95% CrI: 0.24 to 0.89) in 2007. Mean ranks for the tolerability of escitalopram and citalopram were 9.21 and 7.70, respectively, in 2002 and 3.42 and 7.14, respectively, in 2007.

### Relative efficacy and tolerability of escitalopram versus citalopram

**Fig. 6:**
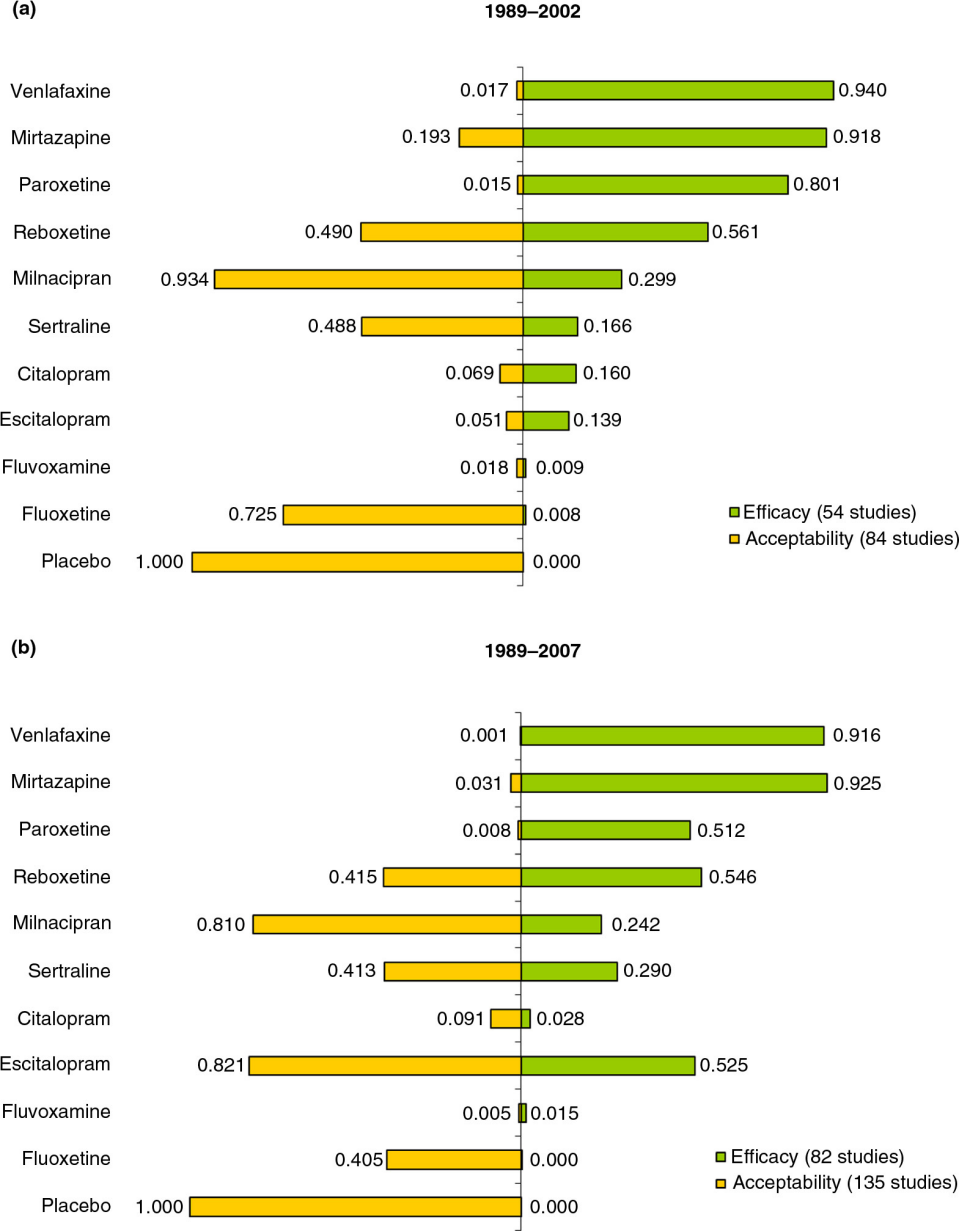
Probability of being ranked among the top four antidepressants for efficacy and tolerability.

The relative efficacy of escitalopram varied considerably over time. In 2002, escitalopram ranked low with just 13.9% and 5.1% chance of being in the top four in terms of relative efficacy and tolerability, respectively. This improved in 2007 when the most favorable balance was for escitalopram with 52.5% probability of being in the top four antidepressants for relative efficacy and 82.1% for tolerability (Fig. 6). By comparison, citalopram continued to have a low probability of being in the top four in terms of both relative efficacy (2.8% in 2007, compared with 16.0% in 2002) and tolerability (9.1% in 2007, compared with 6.9% in 2002) (Fig. 6).

### Relative efficacy and tolerability of all antidepressants

Relative efficacy varied considerably according to the time of the evaluation for several of the other drugs (Fig. 6). Paroxetine exhibited reduced relative efficacy in 2007 compared to 2002, while the relative efficacy of sertraline was enhanced. The tolerability of both of these antidepressants did not change substantially between the two time periods. However, milnacipran, fluvoxamine, and venlafaxine showed little change in efficacy over the two time periods. Milnacipran exhibited a very high tolerability profile and a low efficacy profile for both time periods. Venlafaxine maintained a good efficacy for both time periods, with poor tolerability. In contrast, fluvoxamine presented a low efficacy and tolerability profile during both time periods.

## Discussion and conclusions

This paper reports the results of MTCs that were conducted to examine the relative efficacy and tolerability of a number of new-generation antidepressants in MDD patients between 1989–2002 and 1989–2007. The first time period included registration studies up to time of launch of the reference antidepressant (escitalopram), while the second time period included additional studies that were published up to 5 years later, to coincide with the time of reassessment by HTA authorities several years after the drug was first approved. Despite using the same antidepressants and the same inclusion/exclusion criteria, considerable variation was observed in relative efficacy and tolerability over the two time periods.

In the case of citalopram and escitalopram, both antidepressants had similar efficacy (mean rank 6.14 vs. 6.78) and tolerability (mean rank 7.70 vs. 9.21) around the time of launch for escitalopram (2002). However, greater ability to differentiate between citalopram and escitalopram was observed 5 years later (in 2007) for both efficacy (mean rank 7.27 vs. 4.42) and tolerability (mean rank 7.14 vs. 3.42), with a markedly improved ranking for escitalopram.

Such variability in the relative ranking of antidepressants can arise after a drug has been authorized for the market as the study design of post-marketing trials can differ greatly from the design of the registration trials. Further profiling studies may be conducted post-marketing in patient subgroups in which a new drug is expected to demonstrate a better effect. Subsequent inclusion of data reporting the higher benefits seen in specific patient subgroups for antidepressants may therefore improve ranking of this drug several years after the initial launch date. This is highlighted by the stability of relative efficacy observed for old compounds such as fluvoxamine, for which a low number of studies were conducted after 2002 (Table 1).

The results obtained can also be influenced by shift in treatment guidance and practice, therefore such an overtime comparison requires precautions.

Post-hoc analysis has demonstrated that the benefits of citalopram over placebo are relatively constant, regardless of disease baseline severity, while escitalopram separation from placebo increases with severity of depression (7). As a result, several RCTs designed and published after the approval of escitalopram have shown that escitalopram 20 mg is particularly beneficial in severely depressed patients (baseline MADRS ≥30) (7–9), with superior effects over citalopram or paroxetine reported in this patient subgroup (baseline MADRS ≥30); with the benefits improving further as baseline MADRS score gets higher (7–9). Moreover, while escitalopram 10 mg is the optimal dose for patients with moderate depression (baseline MADRS 22–29), its efficacy decreases in severely depressed patients (10).

These results demonstrate the importance of re-evaluation of a drug over time and have important consequences for HTA agencies and other decision-makers who need to evaluate a drug at its time of launch and make a decision on whether to reimburse the drug. Data presented here on the relative efficacy of a selection of antidepressants during the second time period support a landmark study by Cipriani and colleagues that evaluated the relative efficacy and tolerability of SSRIs and serotonin-noradrenaline reuptake inhibitors (SNRIs) in MDD using NMA based on publications up to 2007 (11). The outcomes of the Cipriani report have since been used by HTA agencies in drug evaluations and clinical guidelines (11, 12). However, the methods used in the Cipriani report have limitations that may influence the outcomes and should be taken into consideration, including bias in study selection (data from placebo-controlled comparisons were excluded if they did not include an active comparator) (13, 14), choice of outcome (the definition of the response may be inconsistent and general withdrawal was considered instead of withdrawal due to AE which is closer to tolerability), and differential drug exposure (further profiling studies in specific patient subpopulations may improve the response of a more established drug, patients that are non-responders/intolerant to a comparator may be excluded, and there may be bias towards response with a more established drug[functional unblinding]) (15). In contrast to the Cipriani study, which included only acute phase studies and head-to-head trials with active comparators, study selection for the MTC analyses reported here also included studies with placebo as the only comparator. Furthermore, the database used for the MTC data reported here included all publications up to 2007 and excluded studies presenting only HAM-D outcomes. Such differences in study selection may explain changes in the ranking of specific antidepressants, such as the improved ranking of reboxetine, compared with the Cipriani report (11).

Typically, any variation in the tolerability of drugs should not be as prominent as for efficacy as it is expected that drugs will have similar tolerability profiles across slightly varying patients’ populations or disease's characteristics. For illustration, variations in tolerability observed in the current analysis may arise from the possibility that patients with improved symptoms, particularly those with severe MDD, will choose not to withdraw from the study despite the onset of AEs. Variations in tolerability may also be associated with important limitations of the MTC methods used, such as untested comparisons between study populations (due to selection/publication bias), pooling of data from several approved drug doses, or the choice of comparator (active reference or placebo) selected for direct and indirect comparisons between antidepressants of interest. These elements can potentially hinder the assumptions of homogeneity and consistency underlying the MTC methods. Thus, although MTCs combining direct and indirect drug comparisons can provide useful information for decision-makers, care must be taken when interpreting findings from such analyses.

No quantitative evaluation of consistency within the presented treatment network has been conducted. However, it is worth noting that no clear sign of inconsistency was identified in the original Cipriani evaluation, and that the results of this are very consistent with what was obtained here on the full time period (up to 2007).

In conclusion, the time of MA may not be the most appropriate time to evaluate the relative efficacy and tolerability of therapies for a number of reasons. First, the comparison of a new drug versus more established agents may be biased due to the limited evidence that has accumulated for newer drugs and the use of potentially sub-optimal doses prior to drug launch. Second, at launch, there may be insufficient time to capture all of the benefits of a new drug and these will not be fully understood until additional data are collected post-registration. Third, current methods of comparison may be limited due to publication or selection bias. As it is important for HTA agencies and decision makers to evaluate a new drug against other alternatives at the time of launch, the recommendation is to compare drugs from studies with very similar experimental conditions in terms of patient populations and study design. One way to ensure this would be to evaluate a new drug against more established drugs based on the data from pivotal studies submitted as part of the registration package at the time of launch (16). Nevertheless, it is clearly important to reassess the drug relative effect several years after its launch date. In this case, appropriate MTCs should be conducted 5 years after drug launch based on all relevant RCTs, when clinical development of the drug is often completed and there is likely to be less variation of drug relative efficacy and relative tolerability over time.

## Appendices

### Appendix A: List of references corresponding to relevant RCTs used in the MTC analyses of the efficacy outcome

1. Aguglia E, Casacchia M, Cassano GB, Faravelli C, Ferrari G, Giordano P, et al. Double-blind study of the efficacy and safety of sertraline versus fluoxetine in major depression. Int Clin Psychopharmacol 1993; 8(3): 197–202.

2. Allard P, Gram L, Timdahl K, Behnke K, Hanson M, Sogaard J. Efficacy and tolerability of venlafaxine in geriatric outpatients with major depression: A double-blind, randomised 6-month comparative trial with citalopram. Int J Geriatr Psychiatry 2004; 19(12): 1123–30.

3. Alves TC, Cachola I, Brandao J. Efficacy and tolerability of venlafaxine and fluoxetine in outpatients with major depression. Prim Care Psychiatry 1999; 5(2): 57–63.

4. Andreoli V, Caillard V, Deo RS, Rybakowski JK, Versiani M. Reboxetine, a new noradrenaline selective antidepressant, is at least as effective as fluoxetine in the treatment of depression. J Clin Psychopharmacol 2002; 22(4): 393–9.

5. Ansseau M, Papart P, Troisfontaines B, Bartholome F, Bataille M, Charles G, et al. Controlled comparison of milnacipran and fluoxetine in major depression. Psychopharmacology 1994; 114(1): 131–7.

6. Baldwin DS, Cooper JA, Huusom AK, Hindmarch I. A double-blind, randomized, parallel-group, flexible-dose study to evaluate the tolerability, efficacy and effects of treatment discontinuation with escitalopram and paroxetine in patients with major depressive disorder. Int Clin Psychopharmacol 2006; 21(3): 159–69.

7. Bielski RJ, Ventura D, Chang CC. A double-blind comparison of escitalopram and venlafaxine extended release in the treatment of major depressive disorder. J Clin Psychiatry 2004; 65(9): 1190–6.

8. Bougerol T, Scotto J-C, Patris M, Strub N, Lemming O, Hopfner Petersen HE. Citalopram and fluoxetine in major depression. Comparison of two clinical trials in a psychiatrist setting and in general practice. Clin Drug Investig 1997; 14(2): 77–89.

9. Boulenger JP, Huusom AK, Florea I, Baekdal T, Sarchiapone M. A comparative study of the efficacy of long-term treatment with escitalopram and paroxetine in severely depressed patients. Curr Med Res Opin 2006; 22(7): 1331–41.

10. Bremner JD, Panagides J. Remergon efficacy in major depressive episode: A randomized, double-blind, placebo and amitriptyline controlled study. Clin Neuropsychopharmacol 1992; 15(1 Pt B): 236.

11. Bremner JD. A double-blind comparison of Org 3770, amitriptyline, and placebo in major depression. J Clin Psychiatry 1995; 56(11): 519–25.

12. Burke WJ, Gergel I, Bose A. Fixed-dose trial of the single isomer SSRI escitalopram in depressed outpatients. J Clin Psychiatry 2002; 63(4): 331–6.

13. Claghorn JL, Lesem MD. A double-blind placebo-controlled study of Org 3770 in depressed outpatients. J Affect Disord 1995; 34(3): 165–71.

14. Clerc G. Antidepressant efficacy and tolerability of milnacipran, a dual serotonin and noradrenaline reuptake inhibitor: A comparison with fluvoxamine. Int Clin Psychopharmacol 2001; 16(3): 145–51.

15. Clerc GE, Ruimy P, Verdeau-Palles J. A double-blind comparison of venlafaxine and fluoxetine in patients hospitalized for major depression and melancholia. The Venlafaxine French Inpatient Study Group. Int Clin Psychopharmacol 1994; 9(3): 139–43.

16. Colonna L, Andersen HF, Reines EH. A randomized, double-blind, 24-week study of escitalopram (10 mg/day) versus citalopram (20 mg/day) in primary care patients with major depressive disorder. Curr Med Res Opin 2005; 21(10): 1659–68.

17. Corrigan MH, Denahan AQ, Wright CE, Ragual RJ, Evans DL. Comparison of pramipexole, fluoxetine, and placebo in patients with major depression. Depress Anxiety 2000; 11(2): 58–65.

18. Costa e Silva J. Randomized, double-blind comparison of venlafaxine and fluoxetine in outpatients with major depression. J Clin Psychiatry 1998; 59(7): 352–7.

19. Cunningham LA, Borison RL, Carman JS, Chouinard G, Crowder JE, Diamond BI, et al. A comparison of venlafaxine, trazodone, and placebo in major depression. J Clin Psychopharmacol 1994; 14(2): 99–106.

20. Cunningham LA. Once-daily venlafaxine extended release (XR) and venlafaxine immediate release (IR) in outpatients with major depression. Venlafaxine XR 208 Study Group. Ann Clin Psychiatry 1997; 9(3): 157–64.

21. De Nayer A, Geerts S, Ruelens L, Schittecatte M, De Bleeker E, Van Eeckhoutte I, et al. Venlafaxine compared with fluoxetine in outpatients with depression and concomitant anxiety. Int J Neuropsychopharmacol 2002; 5(2): 115–20.

22. De Wilde J, Spiers R, Mertens C, Bartholome F, Schotte G, Leyman S. A double-blind, comparative, multicentre study comparing paroxetine with fluoxetine in depressed patients. Acta Psychiatr Scand 1993; 87(2): 141–5.

23. Detke MJ, Wiltse CG, Mallinckrodt CH, McNamara RK, Demitrack MA, Bitter I. Duloxetine in the acute and long-term treatment of major depressive disorder: A placebo- and paroxetine-controlled trial. Eur Neuropsychopharmacol 2004; 14(6): 457–70.

24. Dierick M, Ravizza L, Realini R, Martin A. A double-blind comparison of venlafaxine and fluoxetine for treatment of major depression in outpatients. Prog Neuropsychopharmacol Biol Psychiatry 1996; 20(1): 57–71.

25. Doogan DP, Langdon CJ. A double-blind, placebo-controlled comparison of sertraline and dothiepin in the treatment of major depression in general practice. Int Clin Psychopharmacol 1994; 9(2): 95–100.

26. Ekselius L, von Knorring L, Eberhard G. A double-blind multicenter trial comparing sertraline and citalopram in patients with major depression treated in general practice. Int Clin Psychopharmacol 1997; 12(6): 323–31.

27. Fabre L, Birkhimer LJ, Zaborny BA, Wong LF, Kapik BM. Fluvoxamine versus imipramine and placebo: A double-blind comparison in depressed patients. Int Clin Psychopharmacol 1996; 11(2): 119–27.

28. Fabre LF. A 6-week, double-blind trial of paroxetine, imipramine, and placebo in depressed outpatients. J Clin Psychiatry 1992; 53(Suppl): 40–3.

29. Feighner JP, Cohn JB, Fabre LF Jr, Fieve RR, Mendels J, Shrivastava RK, et al. A study comparing paroxetine placebo and imipramine in depressed patients. J Affect Disord 1993; 28(2): 71–9.

30. Forest Laboratories (2000). Flexible-dose comparison of the safety and efficacy of Lu 26-054 (Escitalopram), citalopram, and placebo in the treatment of major depression. Report No.: SCT-MD-02.

31. Forest Laboratories (2005). Two-week double-blind placebo-controlled study of escitalopram in the treatment of severe major depression. Report No.: SCT-MD-26.

32. Forest Laboratories (2004). Double-blind comparison of the safety and efficacy of escitalopram and fluoxetine in the treatment of fluoxetine nonresponders. Report No.: SCT-MD-21.

33. Forest Laboratories (2001). Fixed-dose comparison of the safety and efficacy of escitalopram and fluoxetine in the treatment of major depressive disorder. Report No.: SCT-MD-16.

34. Forest Laboratories (2004). A double-blind, flexible-dose comparison of escitalopram, sertraline and placebo in the treatment of major depressive disorder (MDD). Report No.: SCT-MD-27.

35. Forest Laboratories (2003). Randomized, double-blind comparison of a fixed dose of escitalopram (10 mg/day) and an optimal dosing regimen of sertraline (50–200 mg/day) in the treatment of major depressive disorder. Report No.: SCT-MD-18.

36. Gagiano C. A double blind comparison of paroxetine and fluoxetine in patients with major depression. Br J Clin Res 1993; 4: 145–52.

37. GlaxoSmithKline (2005). A double-blind, placebo-controlled, fixed-dosage study comparing the efficacy and tolerability of paroxetine CR and citalopram to placebo in the treatment of major depressive disorder with anxiety. Report No.: 29060/785.

38. GlaxoSmithKline (1992). A multicenter, double-blind, placebo-controlled fixed-dose evaluation of four doses of paroxetine. Report No.: 29060/009.

39. GlaxoSmithKline (2006). A multi-centre, randomised, double-blind, parallel-group, placebo- and active-controlled, flexible dose study evaluating the efficacy, safety and tolerability of extended-release bupropion hydrochloride (150–300 mg once daily), extended-release venlafaxine. Report No.: AK130939.

40. Goldstein DJ, Mallinckrodt C, Lu Y, Demitrack MA. Duloxetine in the treatment of major depressive disorder: A double-blind clinical trial. J Clin Psychiatry 2002; 63(3): 225–31.

41. Goldstein DJ, Lu Y, Detke MJ, Wiltse C, Mallinckrodt C, Demitrack MA. Duloxetine in the treatment of depression: A double-blind placebo-controlled comparison with paroxetine. J Clin Psychopharmacol 2004; 24(4): 389–99.

42. Guelfi JD, Ansseau M, Corruble E, Samuelian JC, Tonelli I, Tournoux A, et al. A double-blind comparison of the efficacy and safety of milnacipran and fluoxetine in depressed inpatients. Int Clin Psychopharmacol 1998; 13(3): 121–8.

43. Guelfi JD, Ansseau M, Timmerman L, Korsgaard S. Mirtazapine versus venlafaxine in hospitalized severely depressed patients with melancholic features. J Clin Psychopharmacol 2001; 21(4): 425–31.

44. Halikas J. Org 3770 (mirtazapine) versus trazodone: A placebo controlled trial in depressed elderly patients. Hum Psychopharmacol Clin Exp 1995; 10(Suppl 2): S125–S133.

45. Kasper S, de Swart H, Friis Andersen H. Escitalopram in the treatment of depressed elderly patients. Am J Geriatr Psychiatry 2005; 13(10): 884–91.

46. Kiev A. A double-blind, placebo-controlled study of paroxetine in depressed outpatients. J Clin Psychiatry 1992; 53(Suppl): 27–9.

47. Lepola UM, Loft H, Reines EH. Escitalopram (10–20 mg/day) is effective and well tolerated in a placebo-controlled study in depression in primary care. Int Clin Psychopharmacol 2003; 18(4): 211–17.

48. Lilly (2004). Duloxetine versus placebo in the treatment of major depression. Report No.: F1J-MC-HMAQ.

49. Lilly (2004). Duloxetine versus placebo and paroxetine in the acute treatment of major depression, study group A. Report No.: F1J-MC-HMAY Study Group A.

50. Loo H, Hale A, D'haenen H. Determination of the dose of agomelatine, a melatoninergic agonist and selective 5-HT(2C) antagonist, in the treatment of major depressive disorder: A placebo-controlled dose range study. Int Clin Psychopharmacol 2002; 17(5): 239–47.

51. Massana J, Moller HJ, Burrows GD, Montenegro RM. Reboxetine: A double-blind comparison with fluoxetine in major depressive disorder. Int Clin Psychopharmacol 1999; 14(2): 73–80.

52. McPartlin GM, Reynolds A, Anderson C, Casoy J. A comparison of once-daily venlafaxine XR and paroxetine in depressed outpatients treated in general practice. Prim Care Psychiatry 1988; 4(3): 127–32.

53. Mehtonen OP, Sogaard J, Roponen P, Behnke K. Randomized, double-blind comparison of venlafaxine and sertraline in outpatients with major depressive disorder. Venlafaxine 631 Study Group. J Clin Psychiatry 2000; 61(2): 95–100.

54. Mendels J, Johnston R, Mattes J, Riesenberg R. Efficacy and safety of b.i.d. doses of venlafaxine in a dose-response study. Psychopharmacol Bull 1993; 29(2): 169–74.

55. Montgomery SA, Huusom AK, Bothmer J. A randomised study comparing escitalopram with venlafaxine XR in primary care patients with major depressive disorder. Neuropsychobiology 2004; 50(1): 57–64.

56. Moore N, Verdoux H, Fantino B. Prospective, multicentre, randomized, double-blind study of the efficacy of escitalopram versus citalopram in outpatient treatment of major depressive disorder. Int Clin Psychopharmacol 2005; 20(3): 131–7.

57. Nemeroff CB, Thase ME. A double-blind, placebo-controlled comparison of venlafaxine and fluoxetine treatment in depressed outpatients. J Psychiatr Res 2007; 41(3–4): 351–9.

58. Nyth AL, Gottfries CG, Lyby K, Smedegaard-Andersen L, Gylding-Sabroe J, Kristensen M, et al. A controlled multicenter clinical study of citalopram and placebo in elderly depressed patients with and without concomitant dementia. Acta Psychiatr Scand 1992; 86(2): 138–45.

59. Olie J, Gunn K, Katz E. A double-blind placebo-controlled multicentre study of sertraline in the acute and continuation treatment of major depression. Eur Psychiatry 1997; 12(1): 34–41.

60. Patris M, Bouchard JM, Bougerol T, Charbonnier JF, Chevalier JF, Clerc G, et al. Citalopram versus fluoxetine: A double-blind, controlled, multicentre, phase III trial in patients with unipolar major depression treated in general practice. Int Clin Psychopharmacol 1996; 11(2): 129–36.

61. Perahia DG, Wang F, Mallinckrodt CH, Walker DJ, Detke MJ. Duloxetine in the treatment of major depressive disorder: A placebo- and paroxetine-controlled trial. Eur Psychiatry 2006; 21(6): 367–78.

62. Peselow ED, Filippi AM, Goodnick P, Barouche F, Fieve RR. The short- and long-term efficacy of paroxetine HCl: A. Data from a 6-week double-blind parallel design trial vs. imipramine and placebo. Psychopharmacol Bull 1989; 25(2): 267–71.

63. Pfizer (2005). Sertraline/[S,S]-reboxetine combination versus sertraline and [S,S]-reboxetine monotherapy in major depressive disorder (MDD) in a double-blind, placebo-controlled, eight week study. Report No.: A0501075 (NCT00636246).

64. Rickels K, Amsterdam J, Clary C, Fox I, Schweizer E, Weise C. A placebo-controlled, double-blind, clinical trial of paroxetine in depressed outpatients. Acta Psychiatr Scand Suppl 1989; 350: 117–23.

65. Rickels K, Amsterdam J, Clary C, Fox I, Schweizer E, Weise C. The efficacy and safety of paroxetine compared with placebo in outpatients with major depression. J Clin Psychiatry Suppl 1992; 53: 30–2.

66. Roose SP, Sackeim HA, Krishnan KR, Pollock BG, Alexopoulos G, Lavretsky H, et al. Antidepressant pharmacotherapy in the treatment of depression in the very old: A randomized, placebo-controlled trial. Am J Psychiatry 2004; 161(11): 2050–9.

67. Roth D, Mattes J, Sheehan KH, Sheehan DV. A double-blind comparison of fluvoxamine, desipramine and placebo in outpatients with depression. Prog Neuropsychopharmacol Biol Psychiatry 1990; 14(6): 929–39.

68. Rudolph RL, Fabre LF, Feighner JP, Rickels K, Entsuah R, Derivan AT. A randomized, placebo-controlled, dose-response trial of venlafaxine hydrochloride in the treatment of major depression. J Clin Psychiatry 1998; 59(3): 116–22.

69. Rudolph RL, Feiger AD. A double-blind, randomized, placebo-controlled trial of once-daily venlafaxine extended release (XR) and fluoxetine for the treatment of depression. J Affect Disord 1999; 56(2–3): 171–81.

70. Schone W, Ludwig M. A double-blind study of paroxetine compared with fluoxetine in geriatric patients with major depression. J Clin Psychopharmacol 1993; 13(6 Suppl 2): 34S–9S.

71. Schweizer E, Feighner J, Mandos LA, Rickels K. Comparison of venlafaxine and imipramine in the acute treatment of major depression in outpatients. J Clin Psychiatry 1994; 55(3): 104–8.

72. Sechter D, Vandel P, Weiller E, Pezous N, Cabanac F, Tournoux A. A comparative study of milnacipran and paroxetine in outpatients with major depression. J Affect Disord 2004; 83(2–3): 233–6.

73. Sramek J, Kashin K, Jasinsky O, Kardatzke D, Kennedy S, Cutler N. Placebo-controlled study of ABT-200 versus fluoxetine in the treatment of major depressive disorder. Depression 1995; 3(4): 199–203.

74. Stahl SM. Placebo-controlled comparison of the selective serotonin reuptake inhibitors citalopram and sertraline. Biol Psychiatry 2000; 48(9): 894–901.

75. Thase ME. Efficacy and tolerability of once-daily venlafaxine extended release (XR) in outpatients with major depression. The Venlafaxine XR 209 Study Group. J Clin Psychiatry 1997; 58(9): 393–8.

76. Tignol J. A double-blind, randomized, fluoxetine-controlled, multicenter study of paroxetine in the treatment of depression. J Clin Psychopharmacol 1993; 13(6 Suppl 2): 18S–22S.

77. Tylee A, Beaumont G, Bowden MW, Reynolds A. A double-blind, randomized, 12-week comparison study of the safety and efficacy of venlafaxine and fluoxetine in moderate to severe major depression in general practice. Prim Care Psychiatry 1997; 3(1): 51–8.

78. Tzanakaki M, Guazzelli M, Nimatoudis I, Zissis NP, Smeraldi E, Rizzo F. Increased remission rates with venlafaxine compared with fluoxetine in hospitalized patients with major depression and melancholia. Int Clin Psychopharmacol 2000; 15(1): 29–34.

79. Ventura D, Armstrong EP, Skrepnek GH, Haim EM. Escitalopram versus sertraline in the treatment of major depressive disorder: A randomized clinical trial. Curr Med Res Opin 2007; 23(2): 245–50.

80. Versiani M, Moreno R, Ramakers-van Moorsel CJ, Schutte AJ. Comparison of the effects of mirtazapine and fluoxetine in severely depressed patients. CNS Drugs 2005; 19(2): 137–46.

81. Wade A, Michael LO, Bang HK. Escitalopram 10 mg/day is effective and well tolerated in a placebo-controlled study in depression in primary care. Int Clin Psychopharmacol 2002; 17(3): 95–102.

82. Yevtushenko VY, Belous AI, Yevtushenko YG, Gusinin SE, Buzik OJ, Agibalova TV. Efficacy and tolerability of escitalopram versus citalopram in major depressive disorder: A 6-week, multicenter, prospective, randomized, double-blind, active-controlled study in adult outpatients. Clin Ther 2007; 29(11): 2319–32.

### Appendix B: List of references corresponding to relevant RCTs used in the MTC analyses of the safety outcome

1. Hypericum Depression Trial Study Group. Effect of Hypericum perforatum (St John's wort) in major depressive disorder: A randomized controlled trial. JAMA 2002; 287(14): 1807–14.

2. Aguglia E, Casacchia M, Cassano GB, Faravelli C, Ferrari G, Giordano P, et al. Double-blind study of the efficacy and safety of sertraline versus fluoxetine in major depression. Int Clin Psychopharmacol 1993; 8(3): 197–202.

3. Alves TC, Cachola I, Brandao J. Efficacy and tolerability of venlafaxine and fluoxetine in outpatients with major depression. Prim Care Psychiatry 1999; 5(2): 57–63.

4. Andreoli V, Caillard V, Deo RS, Rybakowski JK, Versiani M. Reboxetine, a new noradrenaline selective antidepressant, is at least as effective as fluoxetine in the treatment of depression. J Clin Psychopharmacol 2002; 22(4): 393–9.

5. Ansseau M, Papart P, Troisfontaines B, Bartholome F, Bataille M, Charles G, et al. Controlled comparison of milnacipran and fluoxetine in major depression. Psychopharmacology 1994; 114(1): 131–7.

6. Baldwin DS, Cooper JA, Huusom AK, Hindmarch I. A double-blind, randomized, parallel-group, flexible-dose study to evaluate the tolerability, efficacy and effects of treatment discontinuation with escitalopram and paroxetine in patients with major depressive disorder. Int Clin Psychopharmacol 2006; 21(3): 159–69.

7. Behnke K, Sogaard J, Martin S, Bauml J, Ravindran AV, Agren H, et al. Mirtazapine orally disintegrating tablet versus sertraline: A prospective onset of action study. J Clin Psychopharmacol 2003; 23(4): 358–64.

8. Benkert O, Szegedi A, Kohnen R. Mirtazapine compared with paroxetine in major depression. J Clin Psychiatry 2000; 61(9): 656–63.

9. Benkert O, Szegedi A, Philipp M, Kohnen R, Heinrich C, Heukels A, et al. Mirtazapine orally disintegrating tablets versus venlafaxine extended release: A double-blind, randomized multicenter trial comparing the onset of antidepressant response in patients with major depressive disorder. J Clin Psychopharmacol 2006; 26(1): 75–8.

10. Bennie EH, Mullin JM, Martindale JJ. A double-blind multicenter trial comparing sertraline and fluoxetine in outpatients with major depression. J Clin Psychiatry 1995; 56(6): 229–37.

11. Bielski RJ, Ventura D, Chang CC. A double-blind comparison of escitalopram and venlafaxine extended release in the treatment of major depressive disorder. J Clin Psychiatry 2004; 65(9): 1190–6.

12. Bjerkenstedt L, Edman GV, Alken RG, Mannel M. Hypericum extract LI 160 and fluoxetine in mild to moderate depression: A randomized, placebo-controlled multi-center study in outpatients. Eur Arch Psychiatry Clin Neurosci 2005; 255(1): 40–7.

13. Bougerol T, Scotto J-C, Patris M, Strub N, Lemming O, Hopfner Petersen HE. Citalopram and fluoxetine in major depression. Comparison of two clinical trials in a psychiatrist setting and in general practice. Clin Drug Investig 1997; 14(2): 77–89.

14. Bremner JD, Panagides J. Remergon efficacy in major depressive episode: A randomized, double-blind, placebo and amitriptyline controlled study. Clin Neuropsychopharmacol 1992; 15(1 Pt B): 236.

15. Bremner JD. A double-blind comparison of Org 3770, amitriptyline, and placebo in major depression. J Clin Psychiatry 1995; 56(11): 519–25.

16. Burke WJ, Gergel I, Bose A. Fixed-dose trial of the single isomer SSRI escitalopram in depressed outpatients. J Clin Psychiatry 2002; 63(4): 331–6.

17. Cassano GB, Conti L, Massimetti G, Mengali F, Waekelin JS, Levine J. Use of a standardized documentation system (BLIPS/BDP) in the conduct of a multicenter international trial comparing fluvoxamine, imipramine, and placebo. Psychopharmacol Bull 1986; 22(1): 52–8.

18. Chouinard G, Saxena B, Belanger MC, Ravindran A, Bakish D, Beauclair L, et al. A Canadian multicenter, double-blind study of paroxetine and fluoxetine in major depressive disorder. J Affect Disord 1999; 54(1–2): 39–48.

19. Claghorn JL. The safety and efficacy of paroxetine compared with placebo in a double-blind trial of depressed outpatients. J Clin Psychiatry 1992; 53(Suppl): 33–5.

20. Claghorn JL, Lesem MD. A double-blind placebo-controlled study of Org 3770 in depressed outpatients. J Affect Disord 1995; 34(3): 165–71.

21. Claghorn JL, Earl CQ, Walczak DD, Stoner KA, Wong LF, Kanter D, et al. Fluvoxamine maleate in the treatment of depression: A single-center, double-blind, placebo-controlled comparison with imipramine in outpatients. J Clin Psychopharmacol 1996; 16(2): 113–20.

22. Clerc G. Antidepressant efficacy and tolerability of milnacipran, a dual serotonin and noradrenaline reuptake inhibitor: A comparison with fluvoxamine. Int Clin Psychopharmacol 2001; 16(3): 145–51.

23. Clerc GE, Ruimy P, Verdeau-Palles J. A double-blind comparison of venlafaxine and fluoxetine in patients hospitalized for major depression and melancholia. The Venlafaxine French Inpatient Study Group. Int Clin Psychopharmacol 1994; 9(3): 139–43.

24. Cohn JB, Wilcox C. A comparison of fluoxetine, imipramine, and placebo in patients with major depressive disorder. J Clin Psychiatry 1985; 46(3 Pt 2): 26–31.

25. Coleman CC, Cunningham LA, Foster VJ, Batey SR, Donahue RM, Houser TL, et al. Sexual dysfunction associated with the treatment of depression: A placebo-controlled comparison of bupropion sustained release and sertraline treatment. Ann Clin Psychiatry 1999; 11(4): 205–15.

26. Coleman CC, King BR, Bolden-Watson C, Book MJ, Segraves RT, Richard N, et al. A placebo-controlled comparison of the effects on sexual functioning of bupropion sustained release and fluoxetine. Clin Ther 2001; 23(7): 1040–58.

27. Corrigan MH, Denahan AQ, Wright CE, Ragual RJ, Evans DL. Comparison of pramipexole, fluoxetine, and placebo in patients with major depression. Depress Anxiety 2000; 11(2): 58–65.

28. Costa e Silva J. Randomized, double-blind comparison of venlafaxine and fluoxetine in outpatients with major depression. J Clin Psychiatry 1998; 59(7): 352–7.

29. Croft H, Settle E Jr, Houser T, Batey SR, Donahue RM, Ascher JA. A placebo-controlled comparison of the antidepressant efficacy and effects on sexual functioning of sustained-release bupropion and sertraline. Clin Ther 1999; 21(4): 643–58.

30. Cunningham LA, Borison RL, Carman JS, Chouinard G, Crowder JE, Diamond BI, et al. A comparison of venlafaxine, trazodone, and placebo in major depression. J Clin Psychopharmacol 1994; 14(2): 99–106.

31. De Nayer A, Geerts S, Ruelens L, Schittecatte M, De Bleeker E, Van Eeckhoutte I, et al. Venlafaxine compared with fluoxetine in outpatients with depression and concomitant anxiety. Int J Neuropsychopharmacol 2002; 5(2): 115–20.

32. De Wilde J, Spiers R, Mertens C, Bartholome F, Schotte G, Leyman S. A double-blind, comparative, multicentre study comparing paroxetine with fluoxetine in depressed patients. Acta Psychiatr Scand 1993; 87(2): 141–5.

33. De Rubeis RJ, Hollon SD, Amsterdam JD, Shelton RC, Young PR, Salomon RM, et al. Cognitive therapy vs medications in the treatment of moderate to severe depression. Arch Gen Psychiatry 2005; 62(4): 409–16.

34. Detke MJ, Wiltse CG, Mallinckrodt CH, McNamara RK, Demitrack MA, Bitter I. Duloxetine in the acute and long-term treatment of major depressive disorder: A placebo- and paroxetine-controlled trial. Eur Neuropsychopharmacol 2004; 14(6): 457–70.

35. Dierick M, Ravizza L, Realini R, Martin A. A double-blind comparison of venlafaxine and fluoxetine for treatment of major depression in outpatients. Prog Neuropsychopharmacol Biol Psychiatry 1996; 20(1): 57–71.

36. Dimidjian S, Hollon SD, Dobson KS, Schmaling KB, Kohlenberg RJ, Addis ME, et al. Randomized trial of behavioral activation, cognitive therapy, and antidepressant medication in the acute treatment of adults with major depression. J Consult Clin Psychol 2006; 74(4): 658–70.

37. Doogan DP, Langdon CJ. A double-blind, placebo-controlled comparison of sertraline and dothiepin in the treatment of major depression in general practice. Int Clin Psychopharmacol 1994; 9(2): 95–100.

38. Dunlop SR, Dornseif BE, Wernicke JF, Potvin JH. Pattern analysis shows beneficial effect of fluoxetine treatment in mild depression. Psychopharmacol Bull 1990; 26(2): 173–80.

39. Fabre L, Birkhimer LJ, Zaborny BA, Wong LF, Kapik BM. Fluvoxamine versus imipramine and placebo: A double-blind comparison in depressed patients. Int Clin Psychopharmacol 1996; 11(2): 119–27.

40. Fabre LF, Abuzzahab FS, Amin M, Claghorn JL, Mendels J, Petrie WM, et al. Sertraline safety and efficacy in major depression: A double-blind fixed-dose comparison with placebo. Biol Psychiatry 1995; 38(9): 592–602.

41. Fava M, Hoog SL, Judge RA, Kopp JB, Nilsson ME, Gonzales JS. Acute efficacy of fluoxetine versus sertraline and paroxetine in major depressive disorder including effects of baseline insomnia. J Clin Psychopharmacol 2002; 22(2): 137–47.

42. Fava M, Alpert J, Nierenberg AA, Mischoulon D, Otto MW, Zajecka J, et al. A double-blind, randomized trial of St John's wort, fluoxetine, and placebo in major depressive disorder. J Clin Psychopharmacol 2005; 25(5): 441–7.

43. Feighner JP, Boyer WF, Merideth CH, Hendrickson GG. A double-blind comparison of fluoxetine, imipramine and placebo in outpatients with major depression. Int Clin Psychopharmacol 1989; 4(2): 127–34.

44. Feighner JP, Cohn JB, Fabre LF Jr, Fieve RR, Mendels J, Shrivastava RK, et al. A study comparing paroxetine placebo and imipramine in depressed patients. J Affect Disord 1993; 28(2): 71–9.

45. Feighner JP, Overo K. Multicenter, placebo-controlled, fixed-dose study of citalopram in moderate-to-severe depression. J Clin Psychiatry 1999; 60(12): 824–30.

46. Forest Laboratories (2005). A double-blind, flexible-dose comparison of escitalopram, sertraline and placebo in the treatment of major depressive disorder (MDD). Report No.: SCT-MD-27.

47. Forest Laboratories (2005). Two-week double-blind placebo-controlled study of escitalopram in the treatment of severe major depression. Report No.: SCT-MD-26.

48. Forest Laboratories (2004). Double-blind comparison of the safety and efficacy of escitalopram and fluoxetine in the treatment of fluoxetine nonresponders. Report No.: SCT-MD-21.

49. Forest Laboratories (2003). Randomized, double-blind comparison of a fixed dose of escitalopram (10 mg/day) and an optimal dosing regimen of sertraline (50–200 mg/day) in the treatment of major depressive disorder. Report No.: SCT-MD-18.

50. Forest Laboratories (2000). Flexible-dose comparison of the safety and efficacy of Lu 26-054 (Escitalopram), citalopram, and placebo in the treatment of major depression. Report No.: SCT-MD-02.

51. Forest Laboratories (2001). Fixed-dose comparison of the safety and efficacy of escitalopram and fluoxetine in the treatment of major depressive disorder. Report No.: SCT-MD-16.

52. Gagiano C. A double blind comparison of paroxetine and fluoxetine in patients with major depression. Br J Clin Res 1993; 4: 145–52.

53. Gastpar M, Singer A, Zeller K. Comparative efficacy and safety of a once-daily dosage of hypericum extract STW3-VI and citalopram in patients with moderate depression: A double-blind, randomised, multicentre, placebo-controlled study. Pharmacopsychiatry 2006; 39(2): 66–75.

54. GlaxoSmithKline (2005). A double-blind, multicentre study to compare the effectiveness and tolerance of paroxetine versus fluvoxamine in depressed patients. Report No.: 29060/112.

55. GlaxoSmithKline (2005). A multicenter, randomized, double-blind, placebo-controlled comparison of paroxetine and fluoxetine in the treatment of major depressive disorder. Report No.: 29060/128.

56. GlaxoSmithKline (2005). A multicenter, double-blind, placebo-controlled comparison of the safety and efficacy and effects on sexual functioning of wellbutrin (bupropion HCL) sustained release (SR) and fluoxetine in outpatients with moderate to severe recurrent major depression. Report No.: WELL AK1A4006.

57. GlaxoSmithKline (1992). A multicenter, double-blind, placebo-controlled fixed-dose evaluation of four doses of paroxetine. Report No.: 29060/009.

58. GlaxoSmithKline (2005). A double-blind, placebo controlled trial to evaluate the clinical effects of immediate release paroxetine and modified release paroxetine in the treatment of major depression. Report No.: 29060/449.

59. GlaxoSmithKline (2005). A double-blind, placebo-controlled, fixed-dosage study comparing the efficacy and tolerability of paroxetine CR and citalopram to placebo in the treatment of major depressive disorder with anxiety. Report No.: 29060/785.

60. GlaxoSmithKline (2005). A double-blind, placebo-controlled study of paroxetine in depressed outpatients. Report No.: 29060/02/001.

61. GlaxoSmithKline (2005). A double-blind, placebo controlled trial to evaluate the clinical effects of immediate release paroxetine and modified release paroxetine in the treatment of major depression. Report No.: 29060/448.

62. GlaxoSmithKline (2006). A double-blind, multicentre study to compare paroxetine and fluoxetine in the treatment of patients with major depressive disorder with regard to antidepressant efficacy, effects on associated anxiety and tolerability. Report No.: 29060/356 (Acute Phase).

63. GlaxoSmithKline (2006). An 8-week, randomized, double-blind, placebo-controlled, multicenter, fixed-dose study comparing the efficacy and safety of a new chemical entity (NCE) or paroxetine to placebo in moderately to severely depressed patients with major depressive disorder. Report No.: NKD20006 (NCT00048204).

64. GlaxoSmithKline (2006). A multi-centre, randomised, double-blind, parallel-group, placebo- and active-controlled, flexible dose study evaluating the efficacy, safety and tolerability of extended-release bupropion hydrochloride (150–300mg once daily), extended-release venlafaxine. Report No.: AK130939.

65. GlaxoSmithKline (2007). A multicenter, double-blind randomized placebo-controlled comparison of the effects on sexual functioning of extended-release bupropion hydrochloride (300–450 mg) and escitalopram (10–20 mg) in outpatients with moderate to severe major depression over an eight-week treatment period. Report No.: AK130927 (NCT00051272).

66. GlaxoSmithKline (2007). A multicenter, double-blind, randomized, placebo controlled comparison of the effects on sexual functioning of extended-release bupropion hydrochloride (300–450 mg) and escitalopram (10–20 mg) in outpatients with moderate to severe major depression over an eight-week treatment period. Report No.: AK130926 (NCT00051259).

67. GlaxoSmithKline (2007). A randomised, double-blind, double-dummy, parallel-group, placebo-controlled, forced dose titration study evaluating the efficacy and safety of a new chemical entity (NCE) and paroxetine in subjects with major depressive disorder. Report No.: NKF100096 (NCT00413023).

68. GlaxoSmithKline (1993). A double-blind, randomized trial of paroxetine versus placebo in patients with depression accompanied by anxiety. Report No.: 29060/251.

69. GlaxoSmithKline (1991). A multicenter, randomized, double-blind, placebo-controlled comparison of paroxetine and fluoxetine in the treatment of major depressive disorder. Report No.: 29060/115.

70. Goldstein DJ, Mallinckrodt C, Lu Y, Demitrack MA. Duloxetine in the treatment of major depressive disorder: A double-blind clinical trial. J Clin Psychiatry 2002; 63(3): 225–31.

71. Goldstein DJ, Lu Y, Detke MJ, Wiltse C, Mallinckrodt C, Demitrack MA. Duloxetine in the treatment of depression: A double-blind placebo-controlled comparison with paroxetine. J Clin Psychopharmacol 2004; 24(4): 389–99.

72. Guelfi JD, Ansseau M, Corruble E, Samuelian JC, Tonelli I, Tournoux A, et al. A double-blind comparison of the efficacy and safety of milnacipran and fluoxetine in depressed inpatients. Int Clin Psychopharmacol 1998; 13(3): 121–8.

73. Guelfi JD, Ansseau M, Timmerman L, Korsgaard S. Mirtazapine versus venlafaxine in hospitalized severely depressed patients with melancholic features. J Clin Psychopharmacol 2001; 21(4): 425–31.

74. Haffmans PM, Timmerman L, Hoogduin CA. Efficacy and tolerability of citalopram in comparison with fluvoxamine in depressed outpatients: A double-blind, multicentre study. The LUCIFER Group. Int Clin Psychopharmacol 1996; 11(3): 157–64.

75. Halikas J. Org 3770 (mirtazapine) versus trazodone: A placebo controlled trial in depressed elderly patients. Hum Psychopharmacol Clin Exp 1995; 10(Suppl 2): S125–33.

76. Hong CJ, Hu WH, Chen CC, Hsiao CC, Tsai SJ, Ruwe FJ. A double-blind, randomized, group-comparative study of the tolerability and efficacy of 6 weeks’ treatment with mirtazapine or fluoxetine in depressed Chinese patients. J Clin Psychiatry 2003; 64(8): 921–6.

77. Kasper S, de Swart H, Friis AH. Escitalopram in the treatment of depressed elderly patients. Am J Geriatr Psychiatry 2005; 13(10): 884–91.

78. Kiev A. A double-blind, placebo-controlled study of paroxetine in depressed outpatients. J Clin Psychiatry 1992; 53(Suppl): 27–9.

79. Kiev A, Feiger A. A double-blind comparison of fluvoxamine and paroxetine in the treatment of depressed outpatients. J Clin Psychiatry 1997; 58(4): 146–52.

80. Lepola UM, Loft H, Reines EH. Escitalopram (10–20 mg/day) is effective and well tolerated in a placebo-controlled study in depression in primary care. Int Clin Psychopharmacol 2003; 18(4): 211–17.

81. Lilly (2004). Duloxetine versus placebo and paroxetine in the acute treatment of major depression, study group A. Report No.: F1J-MC-HMAY Study Group A.

82. Loo H, Hale A, D'haenen H. Determination of the dose of agomelatine, a melatoninergic agonist and selective 5-HT(2C) antagonist, in the treatment of major depressive disorder: A placebo-controlled dose range study. Int Clin Psychopharmacol 2002; 17(5): 239–47.

83. Lydiard RB, Stahl SM, Hertzman M, Harrison WM. A double-blind, placebo-controlled study comparing the effects of sertraline versus amitriptyline in the treatment of major depression. J Clin Psychiatry 1997; 58(11): 484–91.

84. Massana J, Moller HJ, Burrows GD, Montenegro RM. Reboxetine: A double-blind comparison with fluoxetine in major depressive disorder. Int Clin Psychopharmacol 1999; 14(2): 73–80.

85. McPartlin GM, Reynolds A, Anderson C, Casoy J. A comparison of once-daily venlafaxine XR and paroxetine in depressed otpatiets treated in general practice. Prim Care Psychiatry 1988; 4(3): 127–32.

86. Mehtonen OP, Sogaard J, Roponen P, Behnke K. Randomized, double-blind comparison of venlafaxine and sertraline in outpatients with major depressive disorder. Venlafaxine 631 Study Group. J Clin Psychiatry 2000; 61(2): 95–100.

87. Mendels J, Johnston R, Mattes J, Riesenberg R. Efficacy and safety of b.i.d. doses of venlafaxine in a dose-response study. Psychopharmacol Bull 1993; 29(2): 169–74.

88. Montgomery SA, Rasmussen JG, Lyby K, Connor P, Tanghoj P. Dose response relationship of citalopram 20 mg, citalopram 40 mg and placebo in the treatment of moderate and severe depression. Int Clin Psychopharmacol 1992; 6(Suppl 5): 65–70.

89. Moore N, Verdoux H, Fantino B. Prospective, multicentre, randomized, double-blind study of the efficacy of escitalopram versus citalopram in outpatient treatment of major depressive disorder. Int Clin Psychopharmacol 2005; 20(3): 131–7.

90. Nemeroff CB, Ninan PT, Ballenger J, Lydiard RB, Feighner J, Patterson WM, et al. Double-blind multicenter comparison of fluvoxamine versus sertraline in the treatment of depressed outpatients. Depression 1995; 3(4): 163–9.

91. Nemeroff CB, Thase ME. A double-blind, placebo-controlled comparison of venlafaxine and fluoxetine treatment in depressed outpatients. J Psychiatr Res 2007; 41(3–4): 351–9.

92. Newhouse PA, Krishnan KR, Doraiswamy PM, Richter EM, Batzar ED, Clary CM. A double-blind comparison of sertraline and fluoxetine in depressed elderly outpatients. J Clin Psychiatry 2000; 61(8): 559–68.

93. Nierenberg AA, Greist JH, Mallinckrodt CH, Prakash A, Sambunaris A, Tollefson GD, et al. Duloxetine versus escitalopram and placebo in the treatment of patients with major depressive disorder: Onset of antidepressant action, a non-inferiority study. Curr Med Res Opin 2007; 23(2): 401–16.

94. Nyth AL, Gottfries CG, Lyby K, Smedegaard-Andersen L, Gylding-Sabroe J, Kristensen M, et al. A controlled multicenter clinical study of citalopram and placebo in elderly depressed patients with and without concomitant dementia. Acta Psychiatr Scand 1992; 86(2): 138–45.

95. Olie J, Gunn K, Katz E. A double-blind placebo-controlled multicentre study of sertraline in the acute and continuation treatment of major depression. Eur Psychiatry 1997; 12(1): 34–41.

96. Ontiveros A, Garcia-Barriga C. A double-blind, comparative study of paroxetine and fluoxetine in out-patients with depression. Br J Clin Res 1997; 8: 23–32.

97. Patris M, Bouchard JM, Bougerol T, Charbonnier JF, Chevalier JF, Clerc G, et al. Citalopram versus fluoxetine: A double-blind, controlled, multicentre, phase III trial in patients with unipolar major depression treated in general practice. Int Clin Psychopharmacol 1996; 11(2): 129–36.

98. Perahia DG, Wang F, Mallinckrodt CH, Walker DJ, Detke MJ. Duloxetine in the treatment of major depressive disorder: A placebo- and paroxetine-controlled trial. Eur Psychiatry 2006; 21(6): 367–78.

99. Peselow ED, Filippi AM, Goodnick P, Barouche F, Fieve RR. The short- and long-term efficacy of paroxetine HCl: A. Data from a 6-week double-blind parallel design trial vs. imipramine and placebo. Psychopharmacol Bull 1989; 25(2): 267–71.

100. Pfizer (2005). Sertraline/[S,S]-reboxetine combination versus sertraline and [S,S]-reboxetine monotherapy in major depressive disorder (MDD) in a double-blind, placebo-controlled, eight week study. Report No.: A0501075 (NCT00636246).

101. Rapaport M, Coccaro E, Sheline Y, Perse T, Holland P, Fabre L, et al. A comparison of fluvoxamine and fluoxetine in the treatment of major depression. J Clin Psychopharmacol 1996; 16(5): 373–8.

102. Rapaport MH, Schneider LS, Dunner DL, Davies JT, Pitts CD. Efficacy of controlled-release paroxetine in the treatment of late-life depression. J Clin Psychiatry 2003; 64: 1065–74.

103. Reimherr FW, Chouinard G, Cohn CK, Cole JO, Itil TM, LaPierre YD, et al. Antidepressant efficacy of sertraline: A double-blind, placebo- and amitriptyline-controlled, multicenter comparison study in outpatients with major depression. J Clin Psychiatry 1990; 51(Suppl B): 18–27.

104. Rickels K, Amsterdam J, Clary C, Fox I, Schweizer E, Weise C. A placebo-controlled, double-blind, clinical trial of paroxetine in depressed outpatients. Acta Psychiatr Scand Suppl 1989; 350: 117–23.

105. Roose SP, Sackeim HA, Krishnan KR, Pollock BG, Alexopoulos G, Lavretsky H, et al. Antidepressant pharmacotherapy in the treatment of depression in the very old: A randomized, placebo-controlled trial. Am J Psychiatry 2004; 161(11): 2050–9.

106. Rossini D, Serretti A, Franchini L, Mandelli L, Smeraldi E, De Ronchi D, et al. Sertraline versus fluvoxamine in the treatment of elderly patients with major depression: A double-blind, randomized trial. J Clin Psychopharmacol 2005; 25(5): 471–5.

107. Roth D, Mattes J, Sheehan KH, Sheehan DV. A double-blind comparison of fluvoxamine, desipramine and placebo in outpatients with depression. Prog Neuropsychopharmacol Biol Psychiatry 1990; 14(6): 929–39.

108. Rudolph RL, Fabre LF, Feighner JP, Rickels K, Entsuah R, Derivan AT. A randomized, placebo-controlled, dose-response trial of venlafaxine hydrochloride in the treatment of major depression. J Clin Psychiatry 1998; 59(3): 116–22.

109. Rudolph RL, Feiger AD. A double-blind, randomized, placebo-controlled trial of once-daily venlafaxine extended release (XR) and fluoxetine for the treatment of depression. J Affect Disord 1999; 56(2–3): 171–81.

110. Schatzberg A, Roose S. A double-blind, placebo-controlled study of venlafaxine and fluoxetine in geriatric outpatients with major depression. Am J Geriatr Psychiatry 2006; 14(4): 361–70.

111. Schatzberg AF, Kremer C, Rodrigues HE, Murphy GM Jr. Double-blind, randomized comparison of mirtazapine and paroxetine in elderly depressed patients. Am J Geriatr Psychiatry 2002; 10(5): 541–50.

112. Schneider LS, Nelson JC, Clary CM, Newhouse P, Krishnan KR, Shiovitz T, et al. An 8-week multicenter, parallel-group, double-blind, placebo-controlled study of sertraline in elderly outpatients with major depression. Am J Psychiatry 2003; 160(7): 1277–85.

113. Schone W, Ludwig M. A double-blind study of paroxetine compared with fluoxetine in geriatric patients with major depression. J Clin Psychopharmacol 1993; 13(6 Suppl 2): 34S–9S.

114. Schweizer E, Feighner J, Mandos LA, Rickels K. Comparison of venlafaxine and imipramine in the acute treatment of major depression in outpatients. J Clin Psychiatry 1994; 55(3): 104–8.

115. Sechter D, Vandel P, Weiller E, Pezous N, Cabanac F, Tournoux A. A comparative study of milnacipran and paroxetine in outpatients with major depression. J Affect Disord 2004; 83(2–3): 233–6.

116. Shelton RC, Haman KL, Rapaport MH, Kiev A, Smith WT, Hirschfeld RM, et al. A randomized, double-blind, active-control study of sertraline versus venlafaxine XR in major depressive disorder. J Clin Psychiatry 2006; 67(11): 1674–81.

117. Shrivastava RK, Shrivastava SH, Overweg N, Blumhardt CL. A double-blind comparison of paroxetine, imipramine, and placebo in major depression. J Clin Psychiatry 1992; 53(Suppl): 48–51.

118. Silverstone PH, Ravindran A. Once-daily venlafaxine extended release (XR) compared with fluoxetine in outpatients with depression and anxiety. Venlafaxine XR 360 Study Group. J Clin Psychiatry 1999; 60(1): 22–8.

119. Sir A, D'Souza RF, Uguz S, George T, Vahip S, Hopwood M, et al. Randomized trial of sertraline versus venlafaxine XR in major depression: Efficacy and discontinuation symptoms. J Clin Psychiatry 2005; 66(10): 1312–20.

120. Smith WT, Glaudin V. A placebo-controlled trial of paroxetine in the treatment of major depression. J Clin Psychiatry 1992; 53(Suppl): 36–9.

121. Sramek J, Kashin K, Jasinsky O, Kardatzke D, Kennedy S, Cutler N. Placebo-controlled study of ABT-200 versus fluoxetine in the treatment of major depressive disorder. Depression 1995; 3(4): 199–203.

122. Thase ME. Efficacy and tolerability of once-daily venlafaxine extended release (XR) in outpatients with major depression. The Venlafaxine XR 209 Study Group. J Clin Psychiatry 1997; 58(9): 393–8.

123. Tignol J. A double-blind, randomized, fluoxetine-controlled, multicenter study of paroxetine in the treatment of depression. J Clin Psychopharmacol 1993; 13(6 Suppl 2): 18S–22S.

124. Tollefson GD, Bosomworth JC, Heiligenstein JH, Potvin JH, Holman S. A double-blind, placebo-controlled clinical trial of fluoxetine in geriatric patients with major depression. The Fluoxetine Collaborative Study Group. Int Psychogeriatr 1995; 7(1): 89–104.

125. Trivedi MH, Pigotti TA, Perera P, Dillingham KE, Carfagno ML, Pitts CD. Effectiveness of low doses of paroxetine controlled release in the treatment of major depressive disorder. J Clin Psychiatry 2004; 65(10): 1356–64.

126. Tzanakaki M, Guazzelli M, Nimatoudis I, Zissis NP, Smeraldi E, Rizzo F. Increased remission rates with venlafaxine compared with fluoxetine in hospitalized patients with major depression and melancholia. Int Clin Psychopharmacol 2000; 15(1): 29–34.

127. Van Moffaert M, Bartholome F, Cosyns P, De Nayer A, Mertens C. A controlled comparison of sertraline and fluoxetine in acute and continuation treatment of major depression. Hum Psychopharmacol Clin Exp 1995; 10(5): 393–405.

128. Ventura D, Armstrong EP, Skrepnek GH, Haim EM. Escitalopram versus sertraline in the treatment of major depressive disorder: A randomized clinical trial. Curr Med Res Opin 2007; 23(2): 245–50.

129. Versiani M, Moreno R, Ramakers-van Moorsel CJ, Schutte AJ. Comparison of the effects of mirtazapine and fluoxetine in severely depressed patients. CNS Drugs 2005; 19(2): 137–46.

130. Wade A, Michael LO, Bang HK. Escitalopram 10 mg/day is effective and well tolerated in a placebo-controlled study in depression in primary care. Int Clin Psychopharmacol 2002; 17(3): 95–102.

131. Walczak DD, Apter JT, Halikas JA, Borison RL, Carman JS, Post GL, et al. The oral dose-effect relationship for fluvoxamine: A fixed-dose comparison against placebo in depressed outpatients. Ann Clin Psychiatry 1996; 8(3): 139–51.

132. Wernicke JF, Dunlop SR, Dornseif BE, Zerbe RL. Fixed-dose fluoxetine therapy for depression. Psychopharmacol Bull 1987; 23(1): 164–8.

133. Wernicke JF, Dunlop SR, Dornseif BE, Bosomworth JC, Humbert M. Low-dose fluoxetine therapy for depression. Psychopharmacol Bull 1988; 24(1): 183–8.

134. Wyeth (2005). A multicenter, randomized, double-blind, placebo-controlled, parallel-group, flexible-dose study of DVS-233 SR and venlafaxine ER in adult outpatients with major depressive disorder. Report No.: 3151A1.317 (NCT00087737).

135. Yevtushenko VY, Belous AI, Yevtushenko YG, Gusinin SE, Buzik OJ, Agibalova TV. Efficacy and tolerability of escitalopram versus citalopram in major depressive disorder: A 6-week, multicenter, prospective, randomized, double-blind, active-controlled study in adult outpatients. Clin Ther 2007; 29(11): 2319–32.
